# Quantitative assessment of the regenerative and mineralogenic performances of the zebrafish caudal fin

**DOI:** 10.1038/srep39191

**Published:** 2016-12-19

**Authors:** João Cardeira, Paulo J. Gavaia, Ignacio Fernández, Ibrahim Fatih Cengiz, Joana Moreira-Silva, Joaquim Miguel Oliveira, Rui L. Reis, M. Leonor Cancela, Vincent Laizé

**Affiliations:** 1 ProRegeM PhD Programme, Department of Biomedical Sciences and Medicine, University of Algarve, Campus de Gambelas, Faro, Portugal; 2 Centre of Marine Sciences (CCMAR), University of Algarve, Campus de Gambelas, Faro, Portugal; 3 Department of Biomedical Sciences and Medicine, University of Algarve, Campus de Gambelas, Faro, Portugal; 4 3B’s Research Group, Biomaterials, Biodegradables and Biomimetics, University of Minho, Headquarters of the European Institute of Excellence on Tissue Engineering and Regenerative Medicine, Avepark, Parque de Ciência e Tecnologia, Zona Industrial da Gandra, 4805-017 Barco GMR, Portugal; 5 ICVS/3B’s, PT Government Associated Laboratory, Portugal

## Abstract

The ability of zebrafish to fully regenerate its caudal fin has been explored to better understand the mechanisms underlying *de novo* bone formation and to develop screening methods towards the discovery of compounds with therapeutic potential. Quantifying caudal fin regeneration largely depends on successfully measuring new tissue formation through methods that require optimization and standardization. Here, we present an improved methodology to characterize and analyse overall caudal fin and bone regeneration in adult zebrafish. First, regenerated and mineralized areas are evaluated through broad, rapid and specific chronological and morphometric analysis in alizarin red stained fins. Then, following a more refined strategy, the intensity of the staining within a 2D longitudinal plane is determined through pixel intensity analysis, as an indicator of density or thickness/volume. The applicability of this methodology on live specimens, to reduce animal experimentation and provide a tool for *in vivo* tracking of the regenerative process, was successfully demonstrated. Finally, the methodology was validated on retinoic acid- and warfarin-treated specimens, and further confirmed by micro-computed tomography. Because it is easily implementable, accurate and does not require sophisticated equipment, the present methodology will certainly provide valuable technical standardization for research in tissue engineering, regenerative medicine and skeletal biology.

While mammals, in particular humans, have a limited ability to regenerate tissues, other vertebrates, like teleost fish, can fully restore lost or damaged body parts such as heart, retina, kidney, spinal cord, brain and fins^1^,^2^,^3^,^4^,^5^. Understanding how vertebrates regenerate lost tissues and identifying the underlying mechanisms is crucial not only from a basic scientific point of view but also towards the development of novel therapies for a variety of degenerative and traumatic diseases. More specifically, unveiling the complex mechanisms leading to *de novo* bone formation and mineralization is of great importance for skeletal regenerative medicine.

The caudal fin of the zebrafish *Danio rerio* (Hamilton, 1822) is a valuable model system for regenerative biology as fin tissues are organized in a simple architecture^2^ and their restoration can be easily monitored in detail over time. Caudal fin regeneration is also remarkably fast (less than 2 weeks^6^), as compared to limb regeneration in tetrapods^7^ (up to 30 days in adult axolotl^8^).

The zebrafish caudal fin is composed of segmented bony rays (lepidotrichia) spaced by inter-ray mesenchymal tissue and covered with epidermis; it is vascularized, innervated and pigmented^9^,^10^,^11^. Each lepidotrichium is formed by two concave hemirays that define an inner space filled with intra-ray tissue, mainly composed of mesenchymal cells^2^,^5^,^6^. Mineralized lepidotrichia are produced by osteoblasts (also referred to as scleroblasts in the caudal fin) that secrete and maintain the bone matrix^12^. Given its remarkable regenerative potential, the zebrafish caudal fin system has been increasingly used for drug screening and discovery of factors regulating regeneration and *de novo* osteogenesis, particularly mineralogenesis^13^,^14^,^15^,^16^.

Upon surgical amputation (i.e. finectomy), a regenerative program initiates. Three main events can be distinguished: wound healing, blastema formation and regenerative outgrowth^2^,^5^,^6^. This specific type of regeneration (i.e. epimorphosis) relies on the dedifferentiation of cells resting below the amputation plane to form a blastema, an intermediate structure composed of undifferentiated and highly proliferative cells^17^,^18^,^19^. Distal to each fin ray, blastemas gradually form new mature tissue until the restoration of the ray, through a proximal-distal differentiation gradient of the various cell types, including osteoblasts^20^,^21^,^22^.

Several methods have been used to assess fin regeneration, osteogenesis (overall bone formation) and mineralogenesis (mineral deposition within forming bone; an indicator of osteogenesis) in different contexts. For example, the total fin area or length of lepidotrichia 2 and 3^23^, the length or width of segments^13^,^24^, the number of newly mineralized segments within lepidotrichia^24^,^25^ or the relative position or number of bifurcations^14^,^24^. Also, transgenic tools have facilitated the study of osteogenesis by imaging fluorescent proteins expressed under the control of promoters or enhancers of bone marker genes like *runx2, sp7* and *osteocalcin*^17^. Considering that zebrafish populations used for regeneration studies are usually highly heterogeneous in size, age and even strain, a methodology correcting for inter-specimen variability is of uttermost need. Accordingly, a simple, easily applicable and standardized strategy to evaluate and quantify regeneration and mineralization is currently lacking.

In this work we provide a detailed description of the regenerative and *de novo* mineralogenic profiles and of the main events occurring during fin regeneration and lepidotrichia mineralization. Such data is used for the development of a methodology to rapidly and accurately assess the overall regenerative and mineralogenic performances in this system. Regeneration and mineralization are first assessed through specific histomorphometric parameters. Then, a more refined approach considering the pixel intensity of alizarin red stained areas as an indicator of density or thickness/volume of newly mineralized bone is evaluated. Finally, the suitability of the present methodology for the study and identification of compounds with regenerative and/or mineralogenic activities is addressed through proof-of-concept experiments using retinoic acid and warfarin, two molecules with described effects on bone formation^26^,^27^.

## Results and Discussion

### Lepidotrichia formation and mineralization during caudal fin regeneration

**Figure 1:**
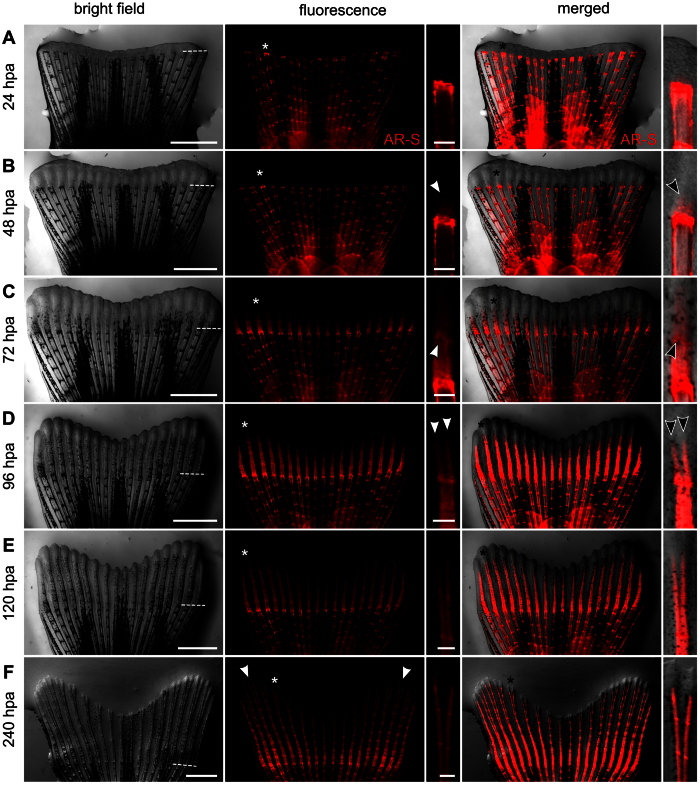
Tissue and lepidotrichia formation during epimorphic regeneration of zebrafish caudal fin at 33 °C. The same specimen was tracked over time and vitally stained with alizarin red S to assess *de novo* mineralization. At appropriate times (24, 48, 72, 96, 120 and 240 hpa), the caudal fin was photographed under bright field and fluorescence illumination. Colour channels were then merged. Single ray images beside fluorescence and merged images are magnifications of the lepidotrichia marked with an asterisk at the corresponding stage. (**A**) at 24 hpa, regenerates were already formed but no mineral deposits were detected. (**B**) first mineral deposits (arrowhead) were detected at 48 hpa. (**C**) at 72 hpa, lepidotrichia were already segmenting as evidenced by the presence of intersegment joints (arrowhead). (**D**) bifurcating lepidotrichia were visible at 96 hpa, with the formation of two new branches at the most distal tip (arrowheads). (**E**) at 120 hpa, formation of the sister rays was well advanced. (**F**) although morphologically regenerated at 240 hpa, zebrafish caudal fins were still undergoing regenerative outgrowth; white arrowheads mark the lepidotrichia number 3 from each fin lobe. In bright field images, dashed lines mark the amputation plane. Scale bar is 1 mm for full fin regenerate images and 100 μm for single ray magnifications.

An accurate assessment of the regenerative and mineralogenic performances of the zebrafish caudal fin requires basic knowledge on the onset and progression of the main events. Hence, a detailed chronological sequence of events was determined through the collection of meristic information from a group of 11 individuals tracked over time (Fig. 1). Individuals were let to regenerate at 33 °C, a commonly used temperature in regeneration studies^16^,^28^,^29^ because it accelerates the whole process^23^,^30^. Mechanisms of fin regeneration have also been studied at 28 °C^31^,^32^,^33^, particularly when dealing with heat shock protein-based gene expression tools. No detailed chronology of regeneration events is yet available at this temperature, although a general delay is expected in the orchestration of the different events.

In the present work, regenerates were observed as soon as 24 hours post-amputation (hpa). However, no mineral deposits could be detected (Fig. 1A; Table 1), in agreement with the observation that most cells of the osteoblastic lineage present near the stump are still in early stages of differentiation^34^. At 48 hpa, when mature extracellular matrix (ECM) secreting osteoblasts are observed^34^, more than half of the regenerates displayed mineral deposits at sites contiguous to the amputation plane and facing the amputated lepidotrichia (Fig. 1B; Table 1). Yet, mineral deposition was low. Its successful detection may depend on the sensitivity of the imaging techniques (e.g. fluorescence *versus* bright-field microscopy) and on the use of demineralizing agents, like acid solutions, before or during staining procedures^35^,^36^,^37^.

The formation of intersegment joints – non-mineralized regions that connect the mineralized bony segments in lepidotrichia^10^ – was observed as early as 72 hpa (Fig. 1C; Table 1), indicating that mechanisms underlying bone remodelling and patterning were already active. The first bifurcating lepidotrichia were observed as early as 96 hpa (Fig. 1D; Table 1) and more than half of the individuals had at least one bifurcated lepidotrichium at 120 hpa (Fig. 1E; Table 1). Bifurcations became more defined as branches continued to mineralize and elongate distally. At 240 hpa, 6 to 10 intersegment joints were visible in the lepidotrichia #3 in each lobe (Fig. 1F; Table 1). From this time on, no additional cellular and structural processes should take place until the conclusion of the regenerative program and attainment of homeostasis^38^.

### Establishing a standardized analytical procedure to assess overall regeneration

**Figure 2:**
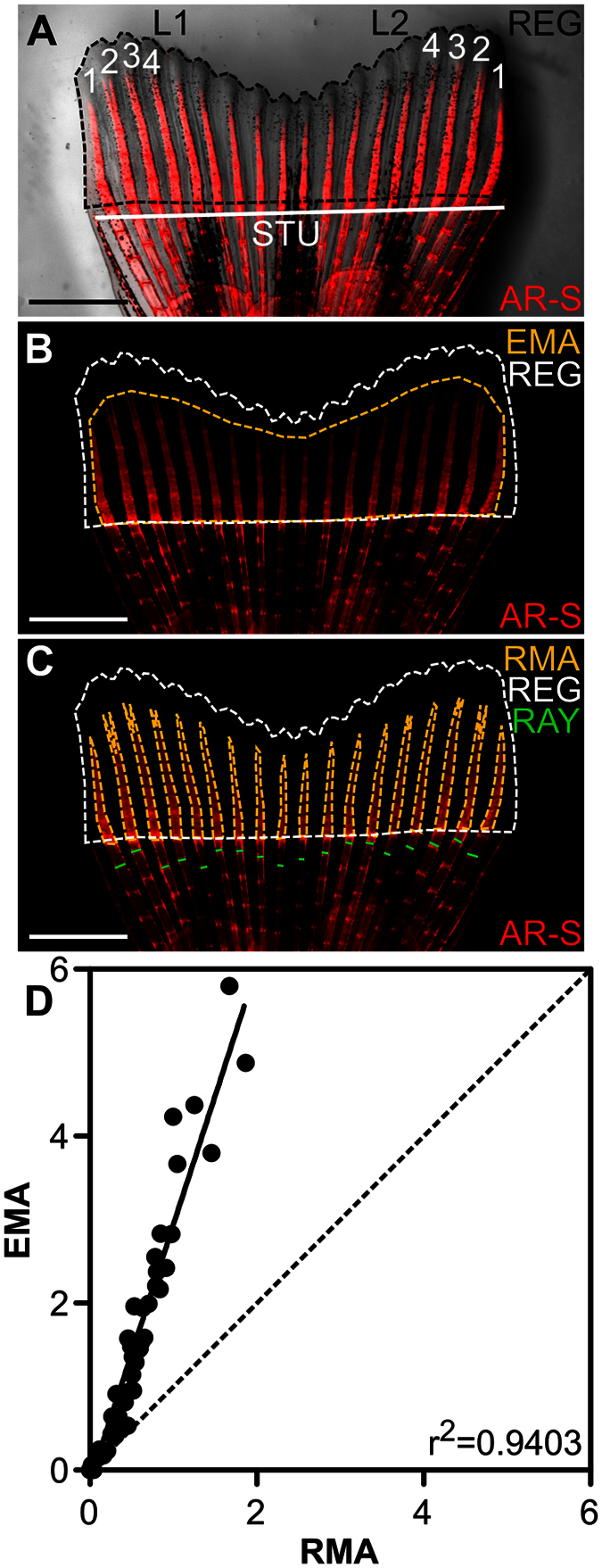
Assessment of the regenerated and mineralized areas. (**A**–**C**) Alizarin red S stained fin at 120 hpa. (**A**) Inter-specimen variability of the regenerated area (REG; outlined by the black dashed line) is corrected with the stump width (STU). Numbers (1–4) show the designation of the lepidotrichia from each of the two fin lobes (L1 and L2). (**B**) Estimated mineralized area (EMA; outlined by the yellow dashed line) corresponds to the area comprising the mineralized lepidotrichia and the inter-ray space. (**C**) Real mineralized area (RMA; outlined by the yellow dashed lines) corresponds to the area stained with alizarin red S, excluding the inter-ray space. The measurements necessary to calculate the mean ray width below the amputation plane (RAY) are also presented. (**D**) Correlation between EMA and RMA is predicted by a simple linear regression (Pearson’s correlation; *P* < 0.0001; *N* = 60).

Fish size and extent of fin regeneration were found to vary within populations used in the course of preliminary experiments, possibly due to natural variations in the physiological condition of fish or to husbandry. Such inter-specimen variation is also expected to occur in other research laboratories, and corrective solutions should be sought to generate reliable and comparable data in this biological system. Fish undergoing fin regeneration were sampled and fixed at regular time-points and the length from the tip of the snout until the amputation plane (LEN; Table 2), the weight (WEI; Table 2) and the stump width (STU; Fig. 2A; Table 2) were determined. The suitability of each of these parameters to correct the regenerated area (REG; Fig. 2A; Table 2) for inter-specimen variability was then evaluated. As expected, REG alone presented a reasonable linear correlation with time (Table 3; Supplementary Fig. S1), but also a high dispersion that gradually increased towards the end of the regenerative process (i.e. 192 hpa onwards; Supplementary Fig. S1). Correcting REG with WEI, LEN or STU resulted in higher linear correlations and decreased data dispersion but to different extents (Table 3; Supplementary Fig. S1). The lowest Pearson’s correlation coefficient was observed for REG/LEN (REG corrected by LEN) and the highest for REG/STU (REG corrected by STU). A high correlation was also observed between STU and LEN or WEI (Supplementary Fig. S1). While LEN, WEI and STU were all appropriate for correcting REG, STU appeared to be the most suitable parameter in a context of medium-throughput screening (i.e. higher data output). It can be rapidly measured from the same micrographs used to determine REG, without the need for additional time-consuming steps (weight and/or total length measurements). Thus, we propose the ratio REG/STU as a standard to normalize and correct the assessment of the regenerated area.

### Establishing a standardized analytical procedure to assess *de novo* mineralization

**Figure 3:**
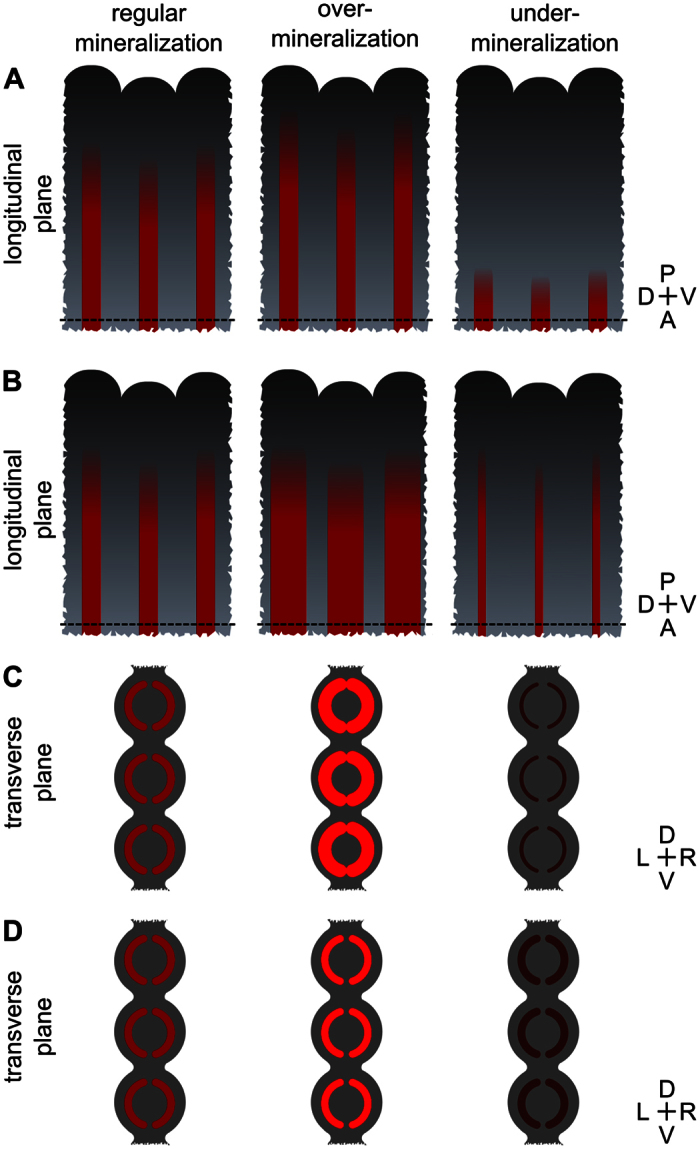
Schematic representation of fin regenerate fragments showing expected mineralogenic outcomes. Mineralogenic effects (either over- or under-mineralization) can occur within the (**A**,**B**) longitudinal or (**C**,**D**) transverse planes. Within the longitudinal plane these effects can occur on (**A**) the anterior-posterior or (**B**) dorsal-ventral directions. Within the transverse plane effects can originate from changes in the (**C**) thickness or (**D**) mineral density of the hemirays. The mineralized rays are represented in red. The different intensities of red refer to (**C**) different ray thickness or (**D**) mineral density, with bright red representing thicker rays or rays with higher mineral density and dark red representing thinner rays or rays with lower mineral density. The dashed lines mark the amputation plane. Orientation of the fin tissue is indicated on the right (P, posterior; A, anterior; L, left; R, right).

Several methods have already been used to assess bone formation during fin regenerating. Some are based on the number of newly mineralized segments in regenerating lepidotrichia^24^,^25^, others have considered segment length or width^13^,^24^, or the relative position or number of bifurcations^14^,^24^. However, these measurements do not directly correlate with the rate of bone formation and mineralization; they are linked to the patterning and not to the extent of newly mineralized tissue. In addition, minor alterations in the rate or degree of mineralization might be easily unnoticed. The possibility of developing a reliable, rapid and direct procedure to quantify the amount of deposited mineral for the identification of potential pro- or anti-mineralogenic agents, either in small trials or large scale screenings, was therefore explored. Extent of mineral deposition can be affected along the anterior-posterior (Fig. 3A) or dorsal-ventral (Fig. 3B) directions within the longitudinal plane, but also through changes in the thickness or volume (Fig. 3C) or mineral density of the rays (Fig. 3D) within the transverse plane. In a first rapid approach, the mineralized areas (determined from fluorescence images after staining with alizarin red S – AR-S) within REG were considered. Two histomorphometric measurements were tested for their suitability in quantifying mineral: i) the estimated mineralized area (EMA; Fig. 2B; Table 2) comprising the mineralized rays and inter-ray spaces and ii) the real mineralized area (RMA; Fig. 2C; Table 2) comprising only the mineralized rays and excluding all the surrounding non-mineralized tissues. A strong linear correlation (*P* < 0.0001) was observed between EMA and RMA (Fig. 2D), showing that both measurements are closely related and suitable to assess the mineralized area. However, by considering the non-mineralized inter-ray spaces, EMA largely overestimates the area of the mineralized fraction of the regenerate (Fig. 2D) and may be less sensitive to small variations in mineral deposition than RMA. In addition, it disregards any dorsal-ventral enlargement or compression (Fig. 3B) of the newly mineralized rays. In that aspect, RMA is more precise and sensitive than EMA and therefore should be used whenever possible, although it implies more laborious procedures. To quickly and easily overcome this issue, ImageJ software was used to automatize RMA measurement through the application of a colour threshold within REG in AR-S fluorescence images. Generally, blue and green values should be set between 0 and 30 and red values should be set up to 255 with a minimum value previously determined for each treatment (expected to be within the range 20–60, according to our data set). It is critical for data homogeneity that i) settings for image acquisition are identical (i.e. exposure time and magnification; gamma set to 1.0; no automatic or manual signal amplification), ii) staining with AR-S is performed simultaneously and with the same solution (precluding eventual differences in exposure to the dye or influence of additional factors in its binding to calcium, such as pH) and iii) the procedure for colour threshold is identical for all RMA measurements. But often, *a priori* intrinsic and individual differences (e.g. background noise due to technical specificities such as microscopy equipment, time of exposure or variable fluorescence within the non-amputated tissue), experimental constraints (e.g. impossibility of staining all specimens at the same time) or the need to compare between different experiments (even though differences should be considered as fold changes relative to the control group), do not permit to maintain the same colour threshold. In those cases, colour threshold should be adjusted to fit RMA between the lateral edges of the rays and the distal mineralized summit. This procedure must be performed methodically and consistently in all samples, preferably by the same analyst in order to minimize the technical bias of RMA measurements. A macro of the semi-automated procedure for RMA measurement in ImageJ software is available in Supplementary Table S2.

The need for minimizing inter-specimen variation in ray width became evident throughout the course of preliminary experiments. While RMA alone exhibited a good linear correlation with time, dividing RMA by the mean ray width at the first inter-segment joint below the amputation plane (RAY; Fig. 2C; Table 2) considerably increased this correlation and reduced data dispersion (Table 3; Supplementary Fig. S3). Hence, we propose the use of RAY as a correcting parameter of RMA.

The mineralogenic effect of a compound can simply be the consequence of an altered regenerative output, thus *de novo* mineralization must be correlated with overall regeneration. Accordingly, effects on the mineralized area, can be masked by effects on the amount of regenerated tissue. It is ultimately relevant to determine the proportion of mineralized tissue within the regenerate, which can be achieved by calculating the ratio (RMA/RAY)/(REG/STU), where RMA and REG are corrected by RAY and STU, respectively. It is worth to mention that RAY weakly correlates with STU (Supplementary Fig. S3), as opposed to WEI and LEN (Supplementary Fig. S3), suggesting that RAY is highly dependent on specimen dimensions but not so much on the length of the amputation plane, further demonstrating the need for adapted correcting factors. In summary, we propose the use of RMA/RAY as a measure of mineral deposition within the regenerate and (RMA/RAY)/(REG/STU) as an estimate of the mineralogenic performance within the context of caudal fin regeneration experiments.

### Modelling and examining the regenerative process in a regular situation

**Figure 4:**
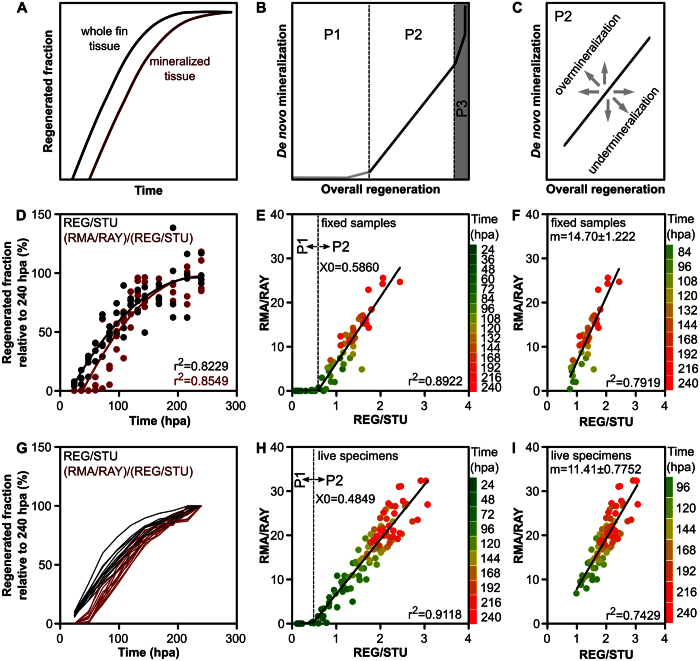
Time-course of caudal fin tissue regeneration and mineralization and correlation between the two processes. (**A–C**) Simplified models designed according to experimental data showing the behaviour of caudal fin regeneration and mineralization (**A**) throughout time and (**B,C**) their correlation. Correlation can be divided into three distinct phases (**B**) where (i) tissue mineralization is not yet effective (P1), (ii) a simple linear regression exists between the tissue regeneration and mineralization (P2) and (iii) tissue regeneration, but not mineralization, is mostly complete (P3; predicted upon additional personal observations). In these models, shifts from the linear regression in P2 would be considered as mineralogenic effects (over- or under-mineralization) (**C**). (**D–F**) Validation of the predicted models using fixed specimens collected at regular intervals. (**D**) Polynomial degree 2 regressions of the whole fin regenerated tissue (REG/STU) and the mineralized fraction of the regenerate ((RMA/RAY)/(REG/STU)) over time (*N* = 60). Each point was converted into a percentage of the mean values at 240 hpa. (**E**) Segmental linear regression of RMA/RAY as a function of REG/STU identifying P1 and P2 (*N* = 60). (**F**) Plot of the data set between 84 and 240 hpa establishing a simple linear correlation between *de novo* mineralization and overall regeneration (Pearson’s correlation; *P* < 0.0001; *N* = 40). (**G–I**) Validation of the predicted models using live specimens vitally stained at regular intervals (*N* = 11). (**G**) Individual tracking of REG/STU and (RMA/RAY)/(REG/STU) over time. For each time-point, the values were converted into a percentage of the value at 240 hpa. (**H**) Segmental linear regression of RMA/RAY as a function of REG/STU identifying P1 and P2 (*N* = 110; 11 specimens analysed at 10 time-points). (**I**) Plot of the data set between 96 and 240 hpa, establishing a simple linear correlation between *de novo* mineralization and overall regeneration (Pearson’s correlation; *P* < 0.0001; *N* = 77). X0 = X value at the intersection between each of the segments of the regression. m = slope. Colour code indicates time (hpa) for each sampling points.

Data presented in Supplementary Figs S1 and S3 suggested that REG/STU and RMA/RAY, and therefore the mineralized fraction of the regenerate, may not increase linearly throughout the entire process. As proposed in the model presented in Fig. 4, bone mineralization does not occur immediately after fin amputation and slows down towards the end of the regenerative process (Fig. 4A). Three phases were predicted (see model in Fig. 4B). P1 is the phase characterized by wound closure, blastema formation, increase of regenerate mass and beginning of outgrowth but still without or with limited mineralization. P2 is the phase during which the mineralized area increases linearly with fin regeneration. P3 is the phase during which lepidotrichia are still mineralizing, but increment in overall fin mass is limited or inexistent (P3 occurs after 240 hpa and is therefore not visible in the data presented here). The linearity of the correlation between mineralization and regeneration in P2 shows that it is the most accurate phase in predicting and evaluating mineralogenic effects. Any data deviating significantly from the standard linear regression in P2 would be indicative of an effect, i.e. over- or under-mineralization depending on the direction of data shift (upwards or downwards, respectively) in relation to the standard line (Fig. 4C). A shift of the data along this line would be indicative of an effect on overall fin regeneration.

In order to test this model, data collected within the scope of the previous experiment – in which 60 fish (4 per time-point) were sampled from 24 to 240 hpa – was analysed. REG/STU and (RMA/RAY)/(REG/STU) were plotted as functions of time and fitted with degree 2 polynomial regressions (Fig. 4D). Similarly, RMA/RAY was plotted as a function of REG/STU and fitted with a segmental linear regression (Fig. 4E). The overall patterns of the fits and delays at the onset of mineralization and regeneration in Fig. 4D and the identification of the phases P1 and P2 in Fig. 4E clearly validated the proposed models. The data set with REG/STU values higher than 0.5860 (X0, the value of X at the intersection of the 2 segments defined by the best fit) and corresponding to time-points higher than 84 hpa were best fitted with a linear regression (Fig. 4F). As modelled in Fig. 4C, any deviation from this linear regression would be indicative of regenerative and/or mineralogenic effects. Based on these data, we propose that screening/evaluation of pro/anti-mineralogenic molecules and compounds with regenerative effects, using the caudal fin regeneration system, should be performed between 84 and 240 hpa. This time-window initiates soon after the formation of the first segments and approximately when (or immediately before) lepidotrichia start bifurcating (Table 1).

### Vital staining and live imaging as alternatives to stained fixed samples

Compounds may affect caudal fin regenerative and mineralogenic performances at precise and narrow time periods, which can differ from one case to another. Testing different exposure times is therefore advisable when screening for molecules with mineralogenic effects. These imply monitoring at multiple time-points, the manipulation and sacrifice of more specimens and consequently the need for larger fish stocks and housing facilities. In this context, we explored the applicability of a recent methodology that allows the tracking of *de novo* bone formation in the same fish over time^37^, in a way that better attends the 3 R’s (replacement, reduction and refinement) guidelines to reduce animal experimentation^39^ and the Directive 2010/63/EU of the European Parliament. Mineralization of lepidotrichia was monitored in the course of fin regeneration in 11 individuals through vital staining with AR-S. Fluorescence signal was captured as for fixed samples although the exposure time had to be extended. The dataset was analysed as for fixed specimens (Fig. 4G–I) either considering data set per fish (Fig. 4G) or per time-point (Fig. 4H,I). As for fixed specimens, the onset of tissue replacement occurred at approximately 24 hpa, shortly followed by the onset of mineralization, and the rate of regeneration slowed down towards the end of the process, eventually reaching a plateau (Fig. 4G). The correlation between RMA/RAY and REG/STU also provided similar results to those from fixed specimens. P1 and P2 were equally identified by a segmental linear regression, although X0 was slightly lower and the rate of regeneration slightly accelerated in the vitally-stained specimens (Fig. 4H). Data set from 96 to 240 hpa (within the linear phase P2, as defined using fixed specimens) was plotted and fitted with a linear regression (Fig. 4I) that exhibited a slope slightly lower than that observed in fixed specimens, which could suggest a modest decrease in the relative mineralized fraction of the regenerates. Differences between the 2 data sets (i.e. fixed and vitally-stained specimens) may be related to fixation and dehydration/rehydration of the fixed samples before staining, steps that are known to trigger artefacts such as tissue shrinking^40^. Nonetheless, potential effects of repetitive anaesthesia and staining procedures must not be ruled out. However, they did not impair regeneration and mineralization, as demonstrated by the normal regenerative outgrowth of the fin, according to a previous report^37^. Overall, this approach was shown to be a suitable alternative to the analysis of fixed samples, allowing a reduction in the number of used individuals. In addition, if the experimental design allows the individualization of the specimens, regeneration can be monitored individually, instead of being treated based on independent values at each sampling time. This provides additional advantages, like the determination of the individual mineralogenic and regenerative rates.

### Validating the power of the established methodology in specific case-studies

**Figure 5:**
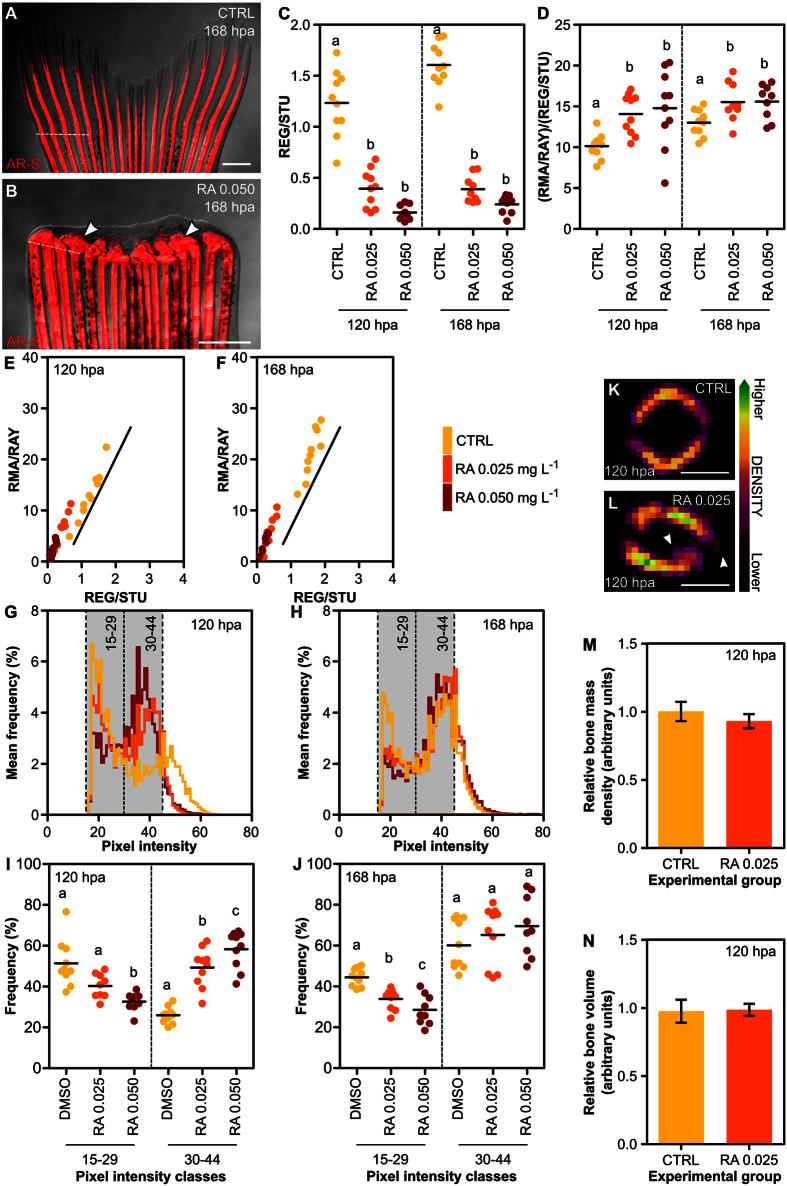
Caudal fin regeneration and mineralization upon exposure to retinoic acid (RA). (**A**,**B**) Regenerating zebrafish caudal fins at 168 hpa exposed to (**A**) DMSO and (**B**) 0.050 mg. L^−1^ of RA. Note the stunted regenerate and fused lepidotrichia (arrowheads). (**C**) Extent of regeneration at 120 and 168 hpa (*P* < 0.0001). (**D**) Extent of mineralization at 120 (*P* < 0.001) and 168 hpa (*P* < 0.05). (**E**,**F**) Mineralization as a function of regeneration and comparison with the standard linear regression (solid line) determined for fixed specimens in Fig. 4, at (**E**) 120 and (**F**) 168 hpa. (**G**,**H**) Distribution of pixel intensity at (**G**) 120 and (**H**) 168 hpa. Grey boxes indicate classes of pixel intensity used in **I** and **J**. (**I**,**J**) Integrated analysis of pixel intensity frequencies (**I**) in classes 15–29 (*P* = 0.0001) and 30–44 (*P* < 0.0001) at 120 hpa and (**J**) in classes 15–29 (*P* < 0.0001) and 30–44 (*P* = 0.3393) at 168 hpa. (**K**,**L**) 2D μ-CT images showing regenerating lepidotrichia number 3 at 120 hpa exposed to (**K**) DMSO and (**L**) 0.025 mg. L^−1^ of RA, evidencing an uneven bone surface with projections into the intra- and extra-ray regions (arrowheads). (**M**,**N**) Relative (**M**) BMD (*P* = 0.4225) and (**N**) bone volume (*P* = 0.9172) in regenerating rays of fins exposed to DMSO and 0.025 mg.L^−1^ of RA at 120 hpa. RA 0.025 = 0.025 mg.L^−1^; RA 0.050 = 0.050 mg.L^−1^; CTRL = DMSO treatments. The mean is represented in scatterplots. Bar graphs show the mean ± SEM. Statistical differences are marked by lower-case letters (a, b and c) for ANOVA (and Tukey’s post hoc) tests. *P* values are indicated above. *N* ≥ 9 for each condition and time-point, except for M and N, in which *N* = 18 (6 rays × 3 fins). Dashed lines mark the amputation planes. Scale bars are 1 mm for A and B and 50 μm for K and L.

**Figure 6:**
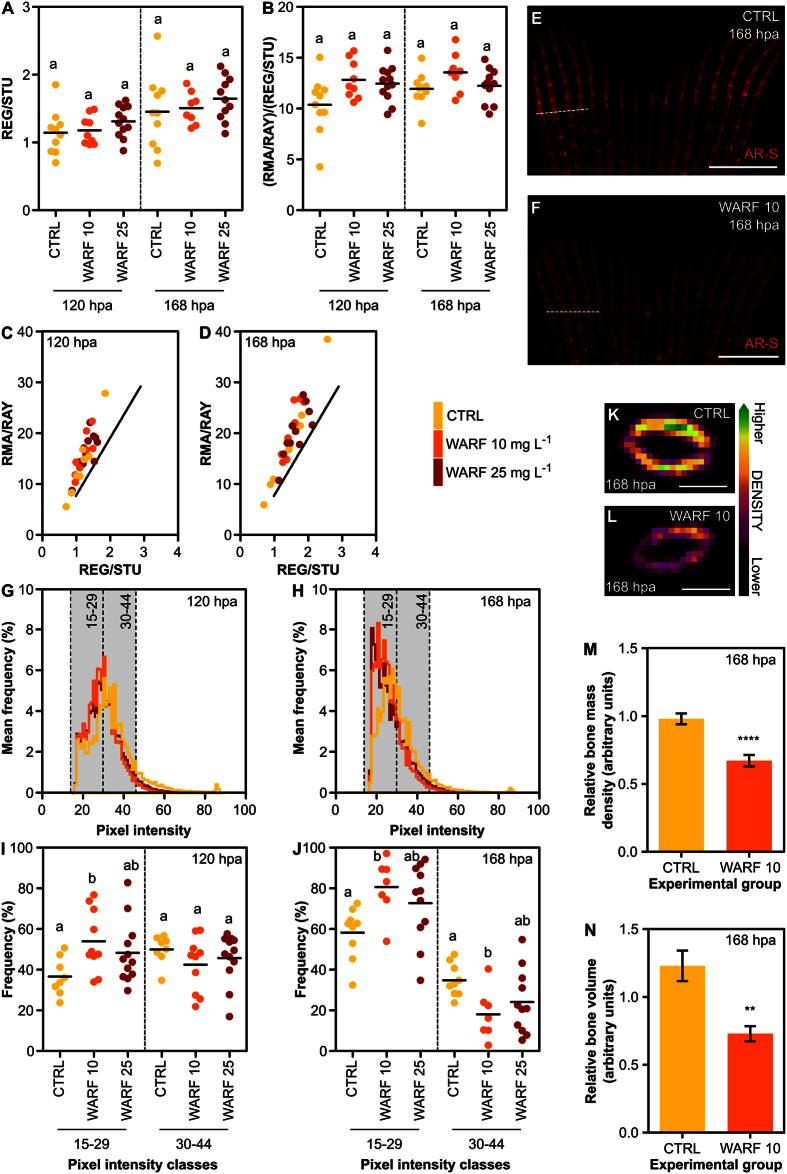
Caudal fin regeneration and mineralization upon exposure to sodium warfarin (WARF). (**A**) Extent of regeneration at 120 (*P* = 0.2971) and 168 hpa (*P* = 0.5302). (**B**) Extent of mineralization at 120 (*P* < 0.05 based on ANOVA; no differences based on Tukey’s test) and 168 hpa (*P* = 0.1383). (**C**,**D**) Mineralization as a function of regeneration and comparison with the standard linear regression (solid line) determined for vitally stained specimens at (**C**) 120 and (**D**) 168 hpa. (**E**,**F**) Regenerating zebrafish caudal fins at 168 hpa exposed to (**E**) vehicle and (**F**) 10 mg. L^−1^ of WARF. Micrographs were not adjusted for colour. (**G**,**H**) Distribution of the pixel intensity at (**G**) 120 and (**H**) 168 hpa. Grey boxes indicate classes of pixel intensity used in **I** and **J**. (**I**,**J**) Integrated analysis of pixel intensity frequencies (**I**) in classes 15–29 (*P* < 0.05) and 30–44 (*P* = 0.4040) at 120 hpa and (**J**) in classes 15–29 (*P* < 0.05) and 30–44 (*P* < 0.05) at 168 hpa. (**K**, **L**) 2D μ-CT images showing regenerating lepidotrichia number 3 at 168 hpa exposed to (**K**) 0 mg. L^−1^ and (**L**) 10 mg. L^−1^ of WARF. (**M**,**N**) Relative (**M**) BMD (****P < 0.0001) and (**N**) bone volume (**P < 0.01) in regenerating rays of fins exposed to 0 and 10 mg.L^−1^ of WARF at 168 hpa. WARF 10 = 10 mg.L^−1^; WARF 25 = 25 mg.L^−1^; CTRL = 0 mg.L^−1^. The mean is represented in scatterplots. Bar graphs show the mean ± SEM. Statistical differences are marked by lower-case letters (a, b and c) or asterisks for ANOVA (and Tukey’s post hoc) or *t* student tests, respectively. *P* values are indicated above. *N* ≥ 9 at 120 hpa and *N* ≥ 7 at 168 hpa for each condition, except for M and N, in which *N* = 18 (6 rays × 3 fins) for CTRL and 12 (6 rays × 2 fins) for WARF 10. Dashed lines mark the amputation planes. Scale bars are 1 mm for E and F and 50 μm for K and L.

Proof-of-concept studies were performed to verify that the rapid area-based procedures described in this work are valid and reliable for identifying pro/anti-regenerative and/or -mineralogenic compounds. Finectomized zebrafish were exposed to different concentrations of retinoic acid (RA; 0.025 and 0.050 mg.L^−1^) and warfarin (WARF; 10 and 25 mg.L^−1^) and their regenerative and mineralogenic performances were compared to that of fish from control groups (Figs 5 and 6). RA-treated fish displayed stunted regenerates with disorganized non-patterned bulks of mineralized tissue showing multiple fusions (Fig. 5A,B). RA has previously been shown to induce fusion of bony elements in the developing vertebral column^41^,^42^ and in the regenerating caudal fin^26^,^43^,^44^,^45^, which has been related to a failure of pre-osteoblasts in respecting ray/inter-ray boundaries^26^. RA exposure was also shown to affect cell proliferation, differentiation and mineralization in two fish skeletal cell lines^46^. In the present study, using fixed specimens, the fin regenerative performance was inhibited in fish exposed to RA, i.e. REG/STU was decreased in treated groups at both 120 and 168 hpa, and the highest concentration had a greater effect (Fig. 5C). RA also induced an increase of the fraction of mineralized tissue within the regenerate (Fig. 5D). At both 120 and 168 hpa, plotted data from RA groups were clearly shifted left and downwards from the control group (which fitted well the linear regression previously determined for fixed specimens), at a position indicative of an under-regenerated and over-mineralized phenotype (Fig. 5E,F). Altogether, previous reports showed that RA levels must be tightly controlled in order to promote optimal overall regeneration, pre-osteoblast proliferation, bone matrix synthesis and balanced osteoclast differentiation^26^,^44^,^45^,^46^,^47^. In the present study, the observed decrease in the regenerative performance may result from an imbalance between pro- and anti-proliferative action of RA on blastema cells, although RA-induced apoptosis in the wound epidermis, as previously reported^43^, cannot be excluded. The present study did not analyse the cellular dynamics of osteoblast populations within the regenerating caudal fin. Even if the proliferative potential of pre-osteoblast populations was affected, it did not impair the production of a mineralized ECM. Regardless of issues on cellular dynamics, our data clearly demonstrated the suitability of the developed procedures to analyse and establish regenerative and mineralogenic effects of RA.

Apart from the extent of mineralized tissue, mineralogenic effects can affect the level of mineral density and/or thickness (from the outer surface to the intra-ray area) and thus volume of newly formed hemirays (Fig. 3C). It is also possible that the pro-mineralogenic action of some agents along the longitudinal plane is limited due to space restrictions within the regenerating tissue. This is particularly relevant if the effects are simple prolongations of the mineralized rays in the anterior-posterior direction (Fig. 3A). Thus, we tested a new approach to evaluate mineralogenic effects within the transverse plane, using the longitudinal plane images collected for the area-based analysis. As it also happens with other fluorescent dyes, like calcein^13^, this approach assumes that AR-S staining (within the longitudinal plane) will be enhanced in high density or thicker-ray areas, thus displaying a stronger fluorescence signal^48^. In this regard, in longitudinal plane images, regions of higher density/thickness would be translated into brighter pixels (i.e. pixels with higher intensity values), while regions of lower density/thickness would be translated into darker pixels (i.e. pixels with lower intensity values). ImageJ software was used to determine the pixel intensity spectrum (frequency distribution of pixel intensity in relation to the total amount of pixels) within the mineralized areas (pixels >15 on the YUV colour model scale). At 120 hpa, AR-S staining of fish exposed to both RA concentrations displayed a peak of pixels with higher intensity (at around 40) than observed in control fish (peak at around 20) (Fig. 5G). At 168 hpa, differences within the pixel intensity histograms between groups were also visible (Fig. 5H), although not as obvious as at 120 hpa. Two classes of pixel intensity – i.e. pixels with values from 15 to 29 and from 30 to 44, designated as low and high intensity pixel classes, respectively – were selected to statistically assess the effects on mineral staining intensity. At 120 hpa, a decrease in low intensity pixels and an increase in high intensity pixels were observed in RA-exposed fish, with the highest concentration displaying a more marked effect, compared to control fish (Fig. 5I). Although a similar tendency was observed at 168 hpa, the differences between control and RA-treated fish were not statistically significant (only for low intensity pixels; Fig. 5J). This data suggests that RA not only increases mineralized area but also enhances mineral deposition itself, such as through increased mineral density and/or thickness of the newly formed lepidotrichia at early/intermediate stages of regeneration. To test this hypothesis, fins at 120 hpa exposed to 0.025 mg.L^−1^ of RA were analysed by micro-computed tomography (μ-CT). Reconstructed 2D images (transverse plane) evidenced a rough, uneven and disorganized bone in RA-treated lepidotrichia in contrast to the smooth and uniform bone surface of the control (Fig. 5K,L), as observed previously in histological sections^44^. Relative bone mineral density (BMD; Fig. 5M) and volume (Fig. 5N) were however unaffected by RA treatment. Given that AR-S binds to calcium^49^, changes in staining intensity might be the result of calcium-specific deposition rather than an overall increase in BMD or bone volume. All the above described results validate the present methodology for the discovery of new pro-mineralogenic molecules.

Warfarin (WARF) is an inhibitor of vitamin K (VK) recycling, routinely used as an anticoagulant drug, as it blocks the γ-carboxylation of VK-dependent proteins (VKDP) like blood clotting factors^50^. The posttranslational γ-carboxylation of VKDPs allows them to bind calcium and control calcium homeostasis. In this regard, WARF also affects VKDPs present in the bone matrix, thus altering bone formation and mineralization^51^,^52^,^53^. Interestingly, *in vitro* studies reported an inhibition of osteoblast differentiation upon WARF exposure^54^, while VK media supplementation promoted osteoblast-to-osteocyte transition and decreased osteoclast differentiation^55^,^56^. Exposure of zebrafish to WARF was found to induce pathological calcification of soft tissues and altered skeletal development during larval stages^27^, and to promote axial skeleton mineralization in an *abcc6a* mutant, while VK inhibited bone matrix mineralization^57^.

In our experimental system, WARF did not alter the regenerative and mineralogenic performances of zebrafish fin, determined through area-based analysis, at both 120 and 168 hpa (Fig. 6A,B). The linear correlation between RMA/RAY and REG/STU further evidenced a similar extent of mineralization in both control and WARF-treated fish (Fig. 6C,D). WARF effects are not as well established as those of RA, for example. This might create a problem when analysing small mineralogenic effects. At this point, we cannot be sure of the sensitivity of this approach (area-based within the longitudinal plane) in detecting such mild mineralogenic outcomes. However, a weaker staining intensity of the regenerates was observed in WARF-treated fish (Fig. 6E,F), suggesting that density or hemi-ray thickness (and thus, volume) may be reduced in newly formed bone upon exposure to warfarin. To test the hypothesis that mineralogenic effects of WARF may occur within the transverse plane in the zebrafish fin, pixel intensity was analysed and an increase in darker pixels at both 120 and 168 hpa and a decrease in brighter pixels at 168 hpa in fish exposed to 10 mg.L^−1^ of WARF were observed (Fig. 6G–J). For reasons that remain to be determined, 25 mg.L^−1^ of WARF had no significant effect on pixel intensity distribution, although a tendency following the pattern observed for 10 mg.L^−1^ of WARF was observed (Fig. 6I,J). The 2D μ-CT analysis indicated a decrease in the BMD and a reduction of hemiray volume in the regenerates of fish treated with 10 mg.L^−1^ of WARF, at 168 hpa (Fig. 6K–N), confirming the suitability of AR-S staining to assess mineralogenic effects in the transverse plane and validating their quantification through the analysis of pixel intensity. Such analysis provided further insights into the mineralogenic effects related to bone density or ray thickness and is, therefore, a complement to other techniques that infer on the organization of the regenerated mineral, such as birefringence analysis^13^,^58^, for the identification of pro- or anti-mineralogenic agents with effects not as evident as those of RA, for example. Our data confirmed the role of WARF in negatively regulating mineral density in zebrafish lepidotrichia, in a way contrasting to that described for the *abcc6a* mutant^57^. This can be simply explained by the fact that those are two distinct mineralizing systems. One takes place during the initial vertebral column development, in a phase characterized by notochord sheath mineralization^59^. The other, occurs during dermal bone regeneration, in which re-differentiated osteoblasts deposit the bone matrix^17^. Moreover, the Abcc6a protein is known to participate in the active transport of drugs into subcellular organelles, possibly impacting on the complex mechanisms underlying tissue mineralization, specifically those involving VK, which could also explain the opposing phenotypes upon WARF exposure. Mutations in the human *ABCC6* gene that have been shown to be associated to pseudoxanthoma elasticum^60^, characterized by calcification of the elastic fibres, further reinforce that the differences between the present work and the previous might be specifically related to the described mutation. Altogether, these results provide evidence of the suitability of this methodology, at least at a refined density-based level, in identifying anti-mineralogenic agents. It also seems to be sensitive enough in detecting mild effects.

## Conclusions

In this work, we proposed a method for investigating regeneration and *de novo* bone matrix mineralization in the zebrafish caudal fin. The main structural events occurring during the regeneration of zebrafish lepidotrichia, i.e. mineralization, segmentation and bifurcation, were characterized in an integrated and chronological manner throughout the regenerative process. This characterization is not only a valuable contribution to the study of the mechanisms underlying epimorphic regeneration but it is also highly relevant to the development of a screening method towards the identification of molecules with regenerative and mineralogenic activities. As a whole, this methodology (summarized in Table 4) points at reducing the impact of inter-specimen variability (through the use of corrective parameters), targeting the time window during which new bone formation linearly correlates with regenerative outgrowth (84 and 240 hpa), limiting animal experimentation (through the use of vital staining) and approaching mineralogenic effects related to bone density or ray thickness/volume (through the analysis of pixel intensity). Proof-of-concept experiments using retinoic acid and warfarin validated the suitability of the proposed methodology to study and unveil new agents with regenerative and mineralogenic/osteogenic activities. It is also an important contribution to the establishment of zebrafish as a tool for drug discovery.

## Materials and Methods

### Ethical statement

All the people involved in animal handling and experimentation received proper training (category B courses accredited by FELASA, the Federation of Laboratory Animal Science Associations) and all fish facilities were accredited by the Portuguese National Authority for Animal Health (DGAV). All experimental procedures involving animals followed the European Directive 2010/63/EU and the related guidelines (European Commission, 2014) and Portuguese legislation (Decreto-Lei 113/2013) for animal experimentation and welfare.

### Caudal fin amputation and regeneration

For all experiments, adult wild-type zebrafish (AB genotype background; ZFIN ID: ZDB-GENO-960809-7), aged 3–4 months, weighting 50–200 mg (mean 100 mg) total weight, and measuring 15–24 mm (mean 19 mm) total length, were used. Caudal fins were amputated 1–2 segments anterior to the bifurcation of the most peripheral branching lepidotrichia. Finectomized fish were allowed to regenerate at 33 (±1) °C at a density of 1 fish per 180 mL, in 900 mL plastic containers (5 specimens per container) filled with water from the rearing system.

### Animal care

Fish were fed twice a day, once with *Artemia* nauplii (EG strain from INVE Aquaculture, Dendermonde, Belgium) and once with commercial dry food (Zebrafeed from Sparos Lda, Olhão, Portugal). Water was renewed (50%) every second day, photoperiod was set to 14 h light and 10 h dark and both pH (7.4 ± 0.2) and conductivity (680 ± 20 μS) were measured regularly and maintained within the range of values defined for zebrafish^61^.

### Time-course of regeneration and mineralization

Caudal fin regeneration and *de novo* mineralization were studied in specimens (60 in total) fixed before analysis. From 24 to 144 h post-amputation (hpa), 4 fish were sampled every 12 h, then every 24 h until 240 hpa. At appropriate times, specimens were given a lethal anaesthesia with tricaine methanesulfonate (MS222; Sigma-Aldrich, St. Louis, MO), photographed, measured (length from the tip of the snout to the amputation plane) and weighted. Regenerated fins were excised from the caudal peduncle and fixed for 24 h in 4% buffered paraformaldehyde (pH 7.4; Sigma-Aldrich) and dehydrated in an increasing ethanol gradient up to 75%, if not further processed immediately. Caudal fins were then processed for mineral staining and detection. In a second trial, 11 un-fixed fish were individually tracked every 24 h from 24 to 240 hpa, through vital mineral staining.

### Mineral staining and imaging

For fixed specimens, mineral in caudal fins was stained for 1 h in a 0.1% alizarin red S (AR-S) and 0.8% potassium hydroxide (KOH; both from Sigma-Aldrich) solution and tissues were cleared in 1% KOH for 24 h. Fins were gradually transferred to glycerol for final clearing, preservation and imaging^35^. For live specimens, fish were immersed for 15 min in a 0.01% AR-S solution (pH 7.4)^37^, anaesthetized in MS222, photographed and returned to their containers. Staining was performed in groups of 5 (in 200 mL of staining solution) or individually (in 100 mL of staining solution), according to the need of tracking the same individuals over time.

All fins were photographed under a MZ 7.5 fluorescence stereomicroscope (Leica Microsystems GmbH, Wetzlar, Germany) coupled to a F-View II camera driven by the Cell^F v2.7 software (Olympus Soft Imaging Solutions GmbH, Münster, Germany). For each fin, bright field and fluorescence images were collected sequentially to assess the regenerated fin and the mineralized area, respectively. Fluorescence micrographs were taken at λ_ex_ = 530–560 nm and λ_em_ = 580 nm with an exposure of 200 ms for fixed specimens and 500 ms for live specimens.

### Drug exposure

Fish were exposed by immersion to all*-trans* retinoic acid (RA; Sigma-Aldrich) from 0 to 168 hpa and to warfarin (WARF; 3-(α-acetonylbenzyl)-4-hydroxycoumarin sodium salt; Sigma-Aldrich) from 2 to 168 hpa (exposure did not start at 0 hpa to avoid fish mortality by bleeding problems due to WARF anti-coagulant properties). Stock solutions (0.025 and 0.050 mg.mL^−1^ for RA; 10 and 25 mg.mL^−1^ for WARF) were prepared in dimethyl sulfoxide (DMSO; Sigma-Aldrich) for RA and MilliQ water (Millipore, Billerica, MA) for WARF, then diluted 1:1000 into 900 mL of system water. Only the vehicle (DMSO or MilliQ water) was added to the control groups. A regular photoperiod was maintained during RA exposure, but the bath was renewed every other day to compensate for RA photodegradation. For each concentration of RA (0.025 and 0.050 mg.L^−1^) and at each time-point (120 and 168 hpa), 10 specimens were processed as described above for fixed specimens. For each concentration of WARF (10 and 25 mg.L^−1^), 10 specimens were tracked over time (at 72, 120 and 168 hpa) and processed as described above for vital staining procedures and imaging. After staining and imaging, a group of selected fins was processed for μ-CT analysis.

### Image analysis and histomorphometry

Fin images were analysed using ImageJ 1.47t (Wayne Rasband, National Institutes of Health, Bethesda, MD) to quantify regeneration and *de novo* mineralization. The total regenerated area (REG; Fig. 2A; Table 2) was measured from the amputation plane distally and corrected with either the stump width (STU; Fig. 2A; Table 2), the weight (WEI; Table 2) or the length from the tip of the snout to the amputation plane (LEN; Table 2). *De novo* mineralization was evaluated from either the estimated mineral area (EMA; Fig. 2B; Table 2), determined by joining the mineralized most distal tips of the mineral in each ray, or the real mineral area (RMA; Fig. 2C; Table 2) determined by applying a colour threshold (RGB colour model) to REG to select only the mineralized red areas. The colour threshold settings were as follows: red was selected from minimum values – not to include background pixels – up to 255; green and blue were both selected from 0 up to 30. Measurements in pixel number were transformed into mm or mm^2^ before calculations using calibrator images. For drug experiments, the intensity of calcium staining was determined from the frequency distribution of the intensity of pixels within the total mineralized areas (between the intensity values 15 and 254 – arbitrary unit – within the YUV colour model). Histograms of pixel intensity were constructed based on the intensity of the pixels within this spectrum which, together with the described set of staining and imaging conditions, excluded non-mineralized areas. Image analysis and histomorphometry were performed by the same analyst.

### Micro-computed tomography (μ-CT) analysis

A number of 3 fins from RA-treated specimens (0.025 mg.L^−1^ at 120 hpa), 2 fins from WARF-treated specimens (10 mg.L^−1^ at 168 hpa) and 3 fins from each of the respective control fish were scanned with a SkyScan 1272 high-resolution μ-CT system (Bruker Micro-CT, Kontich, Belgium). The voltage and current of the X-ray source were set at 60 kV and 166 μA, respectively, and a 0.25-mm thick aluminium filter was used. For each sample, 800 projections with the isotropic voxel size of 3 μm were acquired over a rotation range of 360° with a rotation step of 0.45°. Bone mineral density (BMD) was calculated based on the calibration with two phantoms of known mineral densities (0.25 and 0.75 mg cm^−3^, Bruker Micro-CT) that were scanned under the same conditions as the samples. The cross-sectional images (4904 × 4904 pixels) were reconstructed from the X-ray projections using the NRecon software v1.6.10.2 (Bruker Micro-CT). Bone mineral density (BMD) and volume of the regenerated bone (within the range of 0.1 mm and 0.25 mm above the amputation plane) were determined using the CT Analyser software v1.15.4.0 (Bruker Micro-CT) for lepidotrichia #2, 3 and 4 of each fin lobe (Fig. 2A). For each lepidotrichium, the relative BMD and volumes were calculated by dividing the absolute values by that of the corresponding lepidotrichium in a selected reference fin (from the control groups).

### Statistical analysis

The statistical analyses were performed using Prism v5.00 (GraphPad Software, Inc., La Jolla, CA). The best fit non-linear regressions were calculated as degree 2 polynomial regressions. Linear correlations were calculated assuming a Gaussian distribution of data, according to the Pearson’s correlation coefficient. For group comparisons one-way ANOVA and Tukey’s post hoc tests were applied. For two-group comparisons unpaired Student’s *t* test was applied. Outliers were determined using Grubb’s test (GraphPad Software, Inc.; www.graphpad.com/quickcalcs/Grubbs1.cfm). Significance level was set to α = 0.05 for all tests.

## Additional Information

**How to cite this article:** Cardeira, J. *et al*. Quantitative assessment of the regenerative and mineralogenic performances of the zebrafish caudal fin. *Sci. Rep.* **6**, 39191; doi: 10.1038/srep39191 (2016).

**Publisher's note:** Springer Nature remains neutral with regard to jurisdictional claims in published maps and institutional affiliations.

## Supplementary Material

Supplementary Information

## Figures and Tables

**Table 1:** Key structural steps of caudal fin regeneration placed chronologically throughout the whole process.

Structural steps of regeneration	Hours post amputation									
	24	48	72	96	120	144	168	192	216	240
Formed/forming regenerate (% of individuals)	100	100	100	100	100	100	100	100	100	100
Regenerate with mineral deposits (% of individuals)	0	64	100	100	100	100	100	100	100	100
Mean number of intersegment joints in lepidotrichia #3	0.00	0.00	1.00	2.55	3.73	5.27	5.91	6.45	7.09	7.91
At least one bifurcating lepidotrichium (% of individuals)	0	0	0	45	64	100	100	100	100	100

Regenerate formation, the onset of tissue mineralization, lepidotrichia segmentation and bifurcation were tracked over time in 11 individuals at 24 h intervals from 24 to 240 hpa. *N* = 11.

**Table 2:** Histomorphometric parameters used to assess and correct the regenerated and mineralized areas.

Abbreviation	Measurement	Technical histomorphometric details
REG	Total regenerated area	Full regenerated area, from the amputation plane until the distal extremity of the regenerate
EMA	Estimated mineralized area	Regenerated mineralized area delimited by the distal extremities of the mineralized rays; includes inter-ray tissue
RMA	Real mineralized area	Regenerated mineralized area defined by the outline of the mineralized rays; excludes inter-ray tissue
RAY	Mean ray width	The mean of the width of mineralized rays at the first intersegment joint below the amputation plane
STU	Stump width	The width of the amputation plane
WEI	Total weight	Total weight of the fish
LEN	Total length	Distance from the tip of the snout until the amputation plane

**Table 3:** Correction factors applied to regenerated and mineralized areas.

	Overall regenerated area				Mineralized area	
	REG	REG/WEI	REG/LEN	REG/STU	RMA	RMA/RAY
**Pearson r**	0.7476	0.8464	0.8081	0.8705	0.8491	0.9007
**r**^**2**^	0.5589	0.7164	0.6531	0.7577	0.7210	0.8112
***P*****value**	<0.0001	<0.0001	<0.0001	<0.0001	<0.0001	<0.0001

For regeneration, linear regression was determined between time and a data set left uncorrected or corrected with WEI, LEN or STU. For mineralization, linear regression was determined between time and a data set left uncorrected or corrected with RAY. All linear correlations were determined according to the Pearson’s correlation coefficient, assuming normal data distribution. The corresponding r^2^ and *P* values are depicted.

**Table 4:** Summary of the main procedures to evaluate the regenerative and mineralogenic capacity of given molecules using the zebrafish caudal fin system.

1. General experimental conditions
Amputate caudal fins and expose specimens to the given treatment (either before amputation and/or during regeneration).
Allow specimens to regenerate at the specific conditions of interest (28 or 33 °C).
Perform a standard time-course trial, using the same specific experimental conditions as routinely used.
**2. Staining and imaging**
Collect and fix or *in vivo* image samples at the time-points of interest (between 84 and 240 hpa for trials conducted at 33 °C; an appropriate working time-window should be determined for different temperatures, e.g. 28 °C).
Fixed specimens
Stain for 1 h in a 0.1% AR-S solution prepared in 0.8% KOH.
Clear staining background in 1% KOH.
Live specimens
Stain for 15 min in a 0.01% AR-S solution (pH 7.4).
Wash and anaesthetize.
**3. Analytical procedures**
Using ImageJ (or another imaging software) determine STU, REG, RMA and RAY. RMA can easily be determined within REG, using the colour threshold tool.
Determine REG/STU and RMA/RAY.
As a first approach, correlate RMA/RAY with REG/STU or determine ((RMA/RAY)/(REG/STU)) and evaluate effects on the regenerative and/or mineralogenic performances.
For higher throughputness, values can be plotted against a standard linear regression between REG/STU and RMA/RAY, determined from a standard time-course trial, using points between 84 and 240 hpa.
For higher analytical depth and refinement, analyse for effects on mineralized bone density or thickness/volume by assessing the pixel intensity distribution. First, determine the pixel intensity histogram of the mineralized areas. Second, select classes of pixel intensity and identify differences in the frequency of pixels in each class.

The summary comprises the general methodology, including experimental conditions, staining and imaging for fixed and live specimens and analytical procedures considering regenerated and mineralized areas and mineralized bone density inferred on pixel intensity distribution.
