# Supplementary material for: Quantitative assessment of the regenerative and mineralogenic performances of the zebrafish caudal fin
Source: Sci Rep. 2016 Dec 19;6:39191. doi: 10.1038/srep39191 (PMC5171864; doi:10.1038/srep39191)
Supplement: Supplementary Information [file srep39191-s1.pdf]

Quantitative assessment of the regenerative and mineralogenic performances of the zebrafish caudal fin

João Cardeira, Paulo J. Gavaia, Ignacio Fernández, Ibrahim Fatih Cengiz, Joana Moreira-Silva, Joaquim Miguel Oliveira, Rui Luís Reis, M. Leonor Cancela, Vincent Laizé

SUPPLEMENTARY INFORMATION

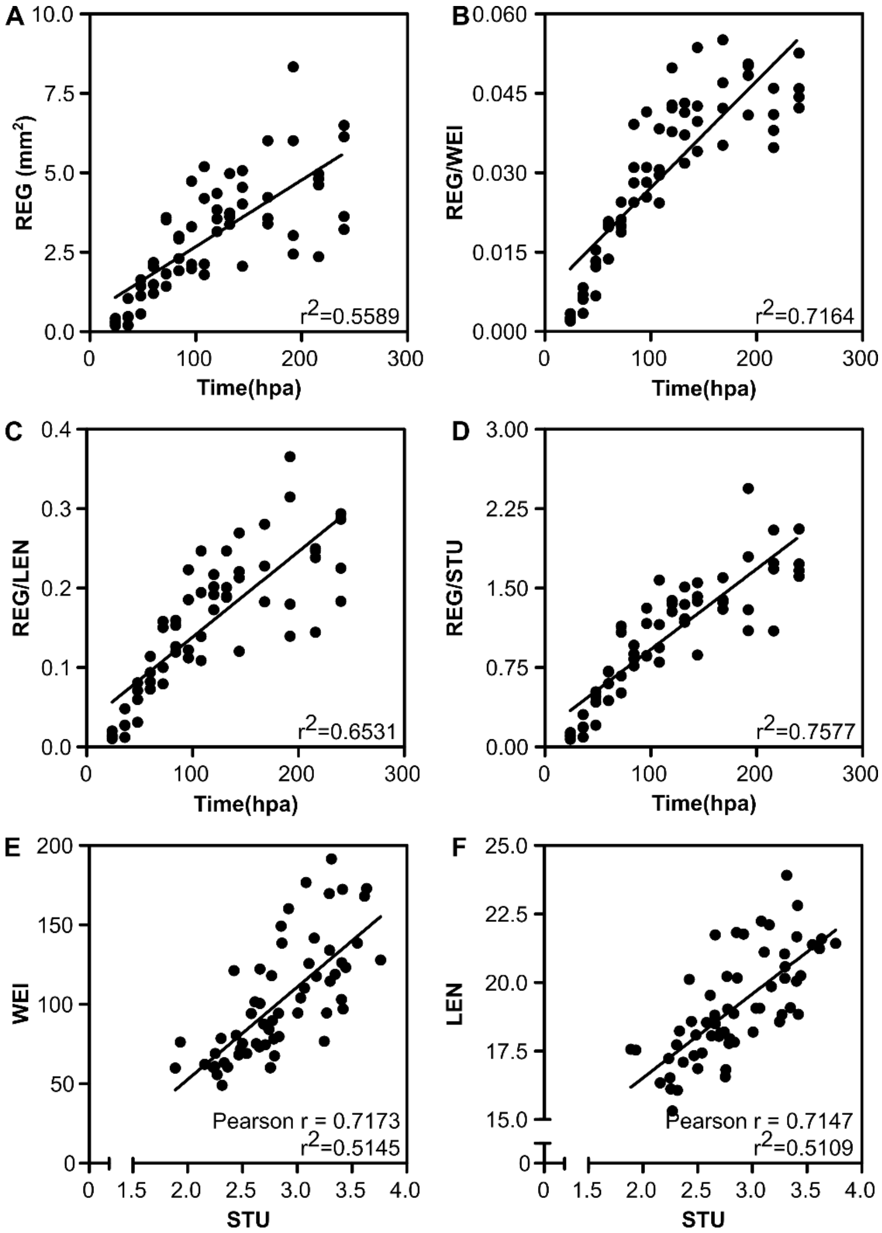

Supplementary Figure S1. Correlation of the regenerated areas with time and between the correcting parameters. (**A**-**D**) Linear regressions between the regenerated area and time, in an (**A**) uncorrected condition and dividing REG by (**B**) WEI, (**C**) LEN and (**D**) STU. (**E**, **F**) Linear regressions and correlations (Pearson's correlation coefficient) between STU and (**E**) WEI and (**F**) LEN. A group of fixed specimens, sampled at regular intervals, was used in all analyses. Pearson's correlations; $P < 0.0001$ for all correlations; $N = 60$.

Supplementary Table S2. ImageJ instructions and simple Macro for the selection of STU, REG, and RMA. The macro can be copied into a basic text processor software and saved in the “Macro” folder within the installed ImageJ software folders, with the file extension .ijm. After manually selecting REG in the bright field micrographs with the Polygon selection tool, run the macro on the correspondent fluorescent micrograph. Select RMA with the colour threshold, according to the RGB colour model and “Measure” the pixel number. In the end, the first line will contain the STU (“Length”), the second will contain the REG (“Area”) and the third will contain RMA (“Area”). All measurements will be retrieved as number of pixels. “Set Measurements” needs to be properly setup before any measurement is performed.

1. Select STU and REG in bright field micrograph;
2. Run the following Macro on the corresponding fluorescent micrograph:
run("Restore Selection");
setBackgroundColor(255, 255, 255);
run("Clear Outside");
run("Measure");
run("Color Threshold...");
3. Select RMA (RGB color model is suggested);
a. Red: Define minimum according to photographs and set maximum to 255;
b. Green: Set minimum and maximum to 0 and 30, respectively;
c. Blue: Set minimum and maximum to 0 and 30, respectively;
4. Copy STU, REG and RMA values into a calculus sheet (e.g. Microsoft Excel).

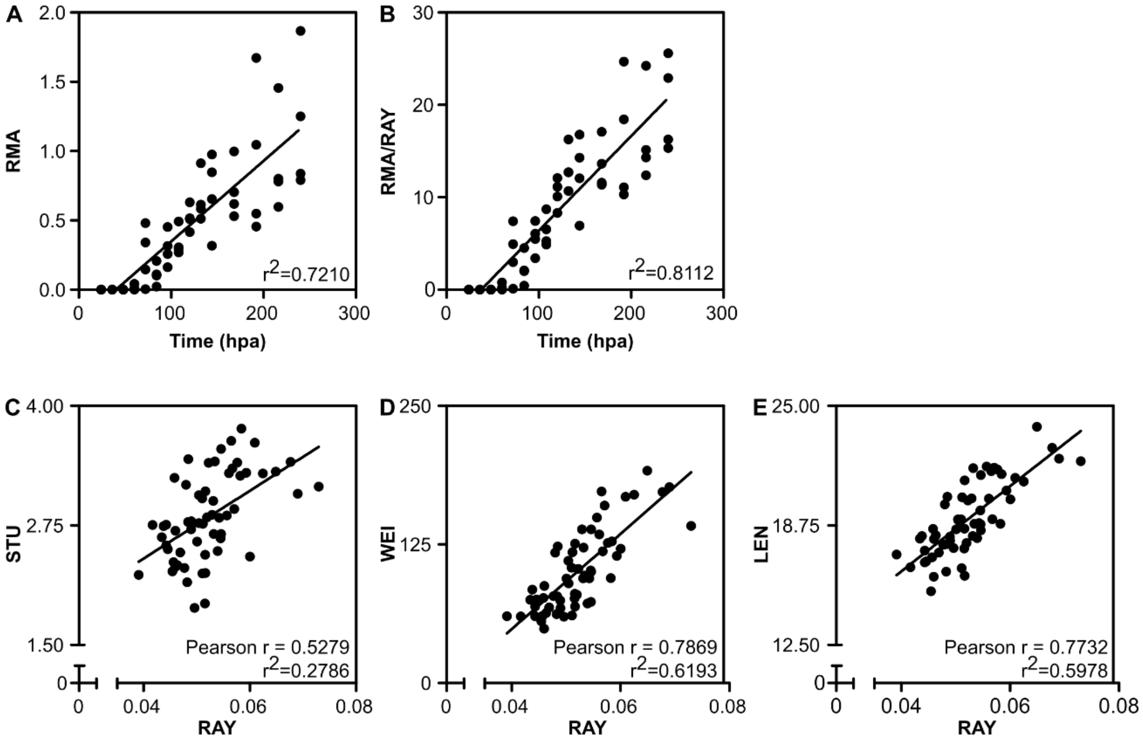

Supplementary Figure S3. Correction of the mineralized area with RAY and correlation of RAY with the correcting parameters of REG. Linear regressions between the mineralized area (RMA) and time, in an (**A**) uncorrected condition and (**B**) dividing RMA by RAY. (**C**-**E**) Linear regressions and correlations between RAY and (**C**) STU, (**D**) WEI and (**E**) LEN. A group of fixed specimens, sampled at regular intervals, was used in all analyses. Pearson's correlations; $P < 0.0001$ for all correlations; $N = 60$.
